# Supplementary material for: Iflavirus Covert Infection Increases Susceptibility to Nucleopolyhedrovirus Disease in Spodoptera exigua
Source: Viruses. 2020 May 5;12(5):509. doi: 10.3390/v12050509 (PMC7290388; doi:10.3390/v12050509)
Supplement: Supplementary file 1 [file viruses-12-00509-s001.zip › Table S1.pdf]

Table 1: Primer sequences and description of the genomic region targeted for qPCR.

Primer	Sequence	Description
SeIV-1q	F: 5′- TGTGAAGTTAGACACGCATGGAA-3′<br>R: 5′-CGACTTGTGCTACTCTCTTCATCAA-3′	Amplifies a 97-bp fragment in the RNA-dependent RNA polymerase (RdRp) region from SeIV-1.
SeIV-2q	F :5′-CCGCTCGCTTATTGAAACGT-3′<br>R: 5′-CATGAGACAGCTGGAATTGGAA-3′	Amplifies a 78-bp fragment in the RNA-dependent RNA polymerase (RdRp) region from SeIV-2.
qATPsynthase	F: 5′-GTTGCTGGTCTGGTGGGATT-3′<br>R: 5′-AGGCCTCAGACACCATTGAAA-3′	Amplifies a 72-bp fragment in ATP-synthase subunit C gene from *S. exigua*.

Carballo, A.; Murillo, R.; Jakubowska, A.; Herrero, S.; Williams, T.; Caballero, P. Co-infection with iflaviruses influences the insecticidal properties of Spodoptera exigua multiple nucleopolyhedrovirus occlusion bodies: Implications for the production and biosecurity of baculovirus insecticides. PLoS One 2017, 12, e0177301:1-14. https://doi.org/10.1371/journal.pone.0177301.t001
