# Supplementary figures and images for: Iflavirus Covert Infection Increases Susceptibility to Nucleopolyhedrovirus Disease in Spodoptera exigua
Source: Viruses. 2020 May 5;12(5):509. doi: 10.3390/v12050509 (PMC7290388; doi:10.3390/v12050509)

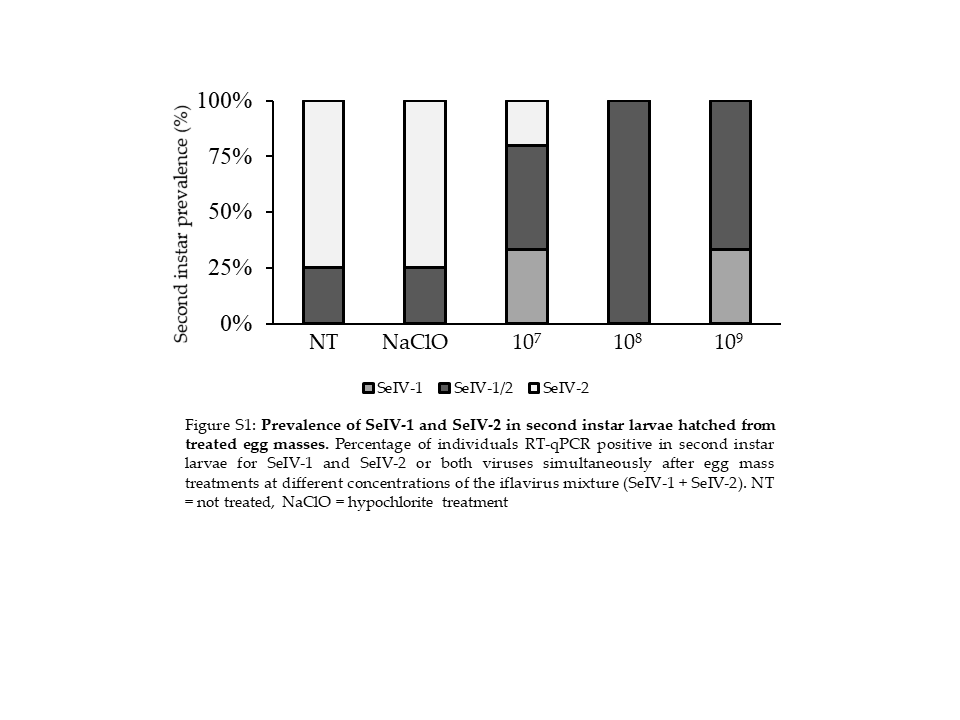

Supplement: Supplementary file 1 [file viruses-12-00509-s001.zip › FigureS1.tif]
